# A Histopathological and Immunohistochemical Analysis of Ameloblastic Fibrodentinoma

**DOI:** 10.1155/2013/604560

**Published:** 2013-02-06

**Authors:** Ronell Bologna-Molina, Sirced Salazar-Rodríguez, Ana María Bedoya-Borella, Ramón Gil Carreón-Burciaga, Gabriel Tapia-Repetto, Nelly Molina-Frechero

**Affiliations:** ^1^Oral Pathology, Research Department, School of Dentistry, Universidad Juárez del Estado de Durango (UJED), 34000 Durango, DGO, Mexico; ^2^School of Dentistry, Universidad de la República (UDELAR), 19200 Montevideo, Uruguay; ^3^Pathology Department, Instituto Nacional de Oncología y Radiobiología (INOR), 10400 Havana, Cuba; ^4^Biology Department, CBC, Universidad de Buenos Aires (UBA), 8000 Buenos Aires, Argentina; ^5^Health Care Department, Universidad Autónoma Metropolitana, Xochimilco, 04960 Mexico City, DF, Mexico

## Abstract

Ameloblastic fibrodentinoma (AFD) is considered a mixed odontogenic tumor that is characterized by conserved epithelial and ectomesenchymal neoplastic components. AFD is composed of long narrow cords and islands of odontogenic epithelium; the epithelial strands lie in a myxoid cell-rich ectomesenchymal tissue with stellate-shaped fibroblasts that exhibit long slender cytoplasmic extensions that resemble dental papilla. The lesions show the presence of dysplastic dentin. Although AFD is a rare entity and its very existence is not completely accepted, based on the extent of histodifferentiation, it is considered to represent a stage between ameloblastic fibroma and ameloblastic fibroodontoma. This study aimed to provide a histopathological and immunohistochemical characterization of this infrequent tumor. A large panel of antibodies including amelogenin, Ck 19, calretinin, syndecan-1, E-cadherin, MSH2, histone H3, and Ki-67 was used to illustrate the nature of the tumor.

## 1. Introduction

Odontogenic tumors (OT) are lesions that are derived from the tooth-producing tissues or their remnants that remain entrapped either within the jawbones or within the adjacent soft tissues. From a biological standpoint, some of these lesions represent hamartomas that exhibit varying degrees of differentiation, whereas others are benign or malignant neoplasms that exhibit variable aggressiveness and a potential to develop metastasis [1].

Ameloblastic Fibrodentinoma (AFD) is considered as “very low frequency” tumor. This rare neoplasm represents less than 1% of all odontogenic tumors in most of the published literature worldwide [1].

Histopathologically, AFD is comprised of an odontogenic ectomesenchyme that resembles the dental papilla and epithelial strands and nests that resemble the dental lamina and enamel organ with the presence of dentin formation.

Occasionally, this odontogenic tumor might be associated with an unerupted tooth, presenting as a slow-growing asymptomatic swelling in the posterior mandible. The age at diagnosis generally falls within the first two decades of life [2].

Treatment consists of enucleation and curettage. Although recurrence is a possibility, it does not justify initial aggressive treatment. AFD rarely progresses to malignancy as ameloblastic fibrodentinosarcoma [3].

The aim of this study was to histopathologically and immunohistochemically characterize this rare tumor using a large panel of antibodies. We furthermore discuss the possible implications or functions that each protein might contribute to the biological behavior of this uncommon tumor.

## 2. Case Report

**Figure 1:**
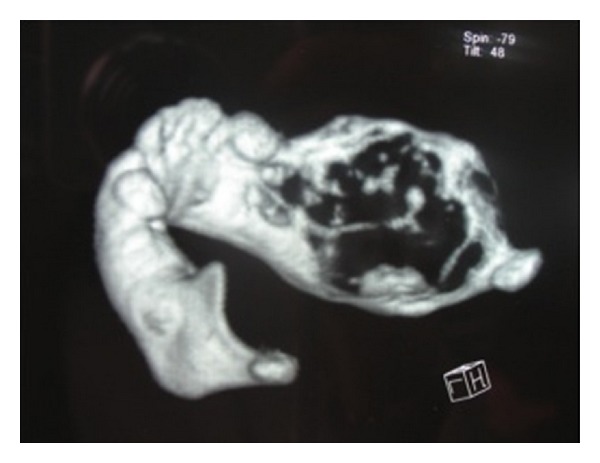
Computed tomography of the mandible showing the extension of the tumor area.

**Figure 2:**
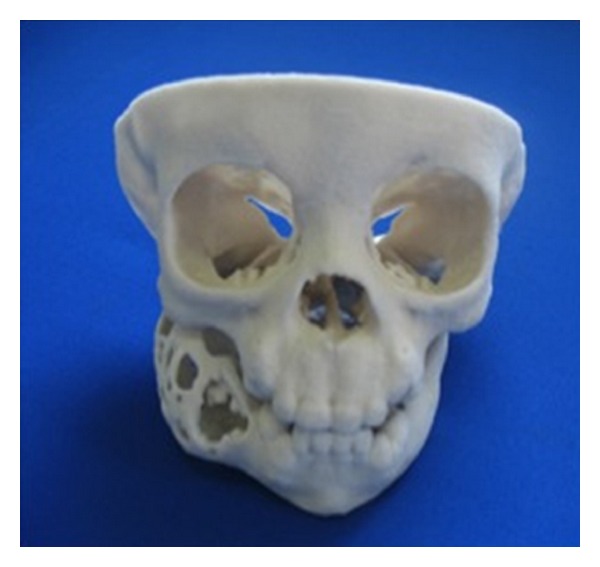
Front view of stereolithography in order to plan surgical treatment.

Female patient, one year and six months old showed evident facial asymmetry and increased volume in the right mandibular body region. In the intraoral examination the patient showed increased volume in the posterior molar region of 6 cm, without pain on palpation and without change of color. Computed tomography (CT) showed an expansive lesion extending from the condyle, covering the ramus and mandibular body (Figure 1). Before conducting the enucleation of the lesion, stereolithography to plan surgical management was conducted (Figure 2). Treatment performed was tumor enucleation and curettage under general anesthesia. The tumor was diagnosed histopathologically as AFD.

## 3. Materials and Methods

The immunohistochemical study conducted two samples from the same patient. Paraffin blocks were sliced into 2 mm thick sections, which were mounted onto polylysine-coated glass slides and were air-dried overnight at room temperature. After deparaffinization and rehydration, the tissue sections were treated with 0.1 M sodium citrate (pH 6.2) and Tween-20 to expose the epitopes. The endogenous peroxidases were blocked with 0.9% hydrogen peroxide, followed by incubation in 1% bovine serum albumin diluted in PBS for 5 min to eliminate nonspecific binding. The sections were incubated with primary antibodies for 45 min. The monoclonal antibodies used are shown in Table 1. After incubation with the primary antibodies, the sections were incubated with biotinylated anti-mouse/rabbit antibodies and with the streptavidin/peroxidase complex for 30 min each (LSAB *þ* labeled streptavidin biotin, Dako). To visualize the reaction, a 3,3′-diaminobenzidine H2O (Dako) substrate was applied. Then, the sections were counterstained with Mayer's hematoxylin solution. For the negative controls, the primary antibody was substituted with PBS.

## 4. Results

The antibodies used to detect the expression of the indicated proteins in the epithelial and mesenchymal cells are shown in Table 1.

## 5. Discussion

Ameloblastic fibromas, ameloblastic fibrodentinomas (AFDs), ameloblastic fibroodontomas and odontomas are lesions that exhibit similar histopathological, clinical, and radiographical features, resulting in a controversial debate over whether they can be delineated as distinct pathological entities or as developmental stages of the same lesion. Some researchers and clinicians consider them as separate entities, whereas others view them as chronological stages of the same lesion, with ameloblastic fibromas at one extreme and odontomas at the other extreme and with ameloblastic fibroodontomas and AFDs in an intermediate stage [4].

The ameloblastic fibromas, AFDs, and ameloblastic fibroodontomas are considered mixed odontogenic tumors that are characterized by conserved epithelial and ectomesenchymal neoplastic components. They are distinguished by the fact that AFDs exhibit dysplastic or tubular dentin, whereas the ameloblastic fibroodontomas exhibit enamel matrix deposits or mature enamel, and ameloblastic fibromas exhibit any type of dental hard tissue deposits. AFD is a rare entity, and its very existence is not completely accepted. Indeed, AFD has been considered to represent a histologically distinct stage between ameloblastic fibroma and ameloblastic fibroodontoma based on the extent of histodifferentiation [5–7].

Currently, whether this lesion represents a separate entity remains unclear. Notably, in the revised WHO classification of odontogenic tumors, ameloblastic fibromas and AFDs are synonymously used terms and are categorized together [3].

Ameloblastic fibromas and AFDs have been suggested to occur in two histologically indistinguishable variants. The first is a neoplastic lesion, which if left in situ does not appear to differentiate further. The second variant is a nonneoplastic, hamartomatous lesion, which appears to be capable of developing into an ameloblastic fibroodontoma and then differentiating further into a complex odontoma [2, 8].

When analyzed microscopically, we observed long narrow cords and islands of odontogenic epithelium. The epithelial strands resided in a myxoid cell-rich stroma with stellate-shaped fibroblasts exhibiting long slender cytoplasmic extensions that resembled dental papilla. The lesions exhibited calcifying elements (dentin matrix and dentinoid material).

**Figure 3:**
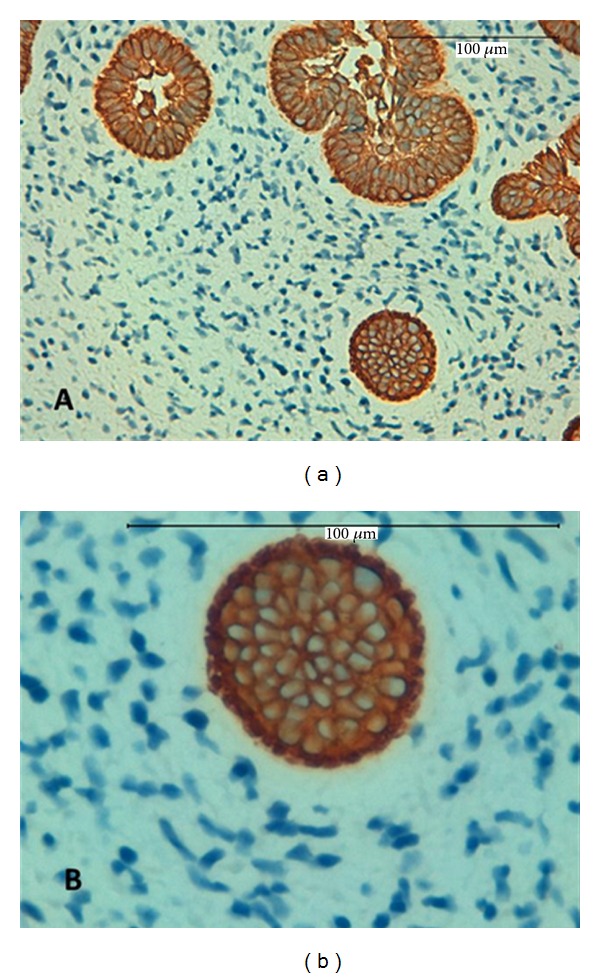
Strong expression for CK19 in the islands and strands of odontogenic epithelium, magnification 400x (a) and 600x (b).

In the immunohistochemical findings of this study, the cords and islands of epithelial cells were also strongly positive for antihuman cytokeratin 19 (CK19) (Figure 3). Cytokeratins (CKs) are the specific intermediate filaments of epithelial cells. They comprise a complex family of at least 20 different polypeptides. The immunoexpression patterns of CKs differ according to cell type, developmental stage, differentiation status, and anatomical site [9]. Various CKs are expressed in tooth germ tissue. However, CK19 is expressed in all types of odontogenic epithelial cells in the developing tooth germ [10] and in neoplastic epithelial cells in some odontogenic tumors [11–13].

The strong immunopositivity that we found for CK19 in the epithelial cells confirms the odontogenic nature of this lesion.

Amelogenins (AMELs) represent the main family of proteins secreted by ameloblasts during amelogenesis (90% of enamel proteins) [14]. AMELs are cell adhesion proteins that play a role in the biomineralisation of teeth. They regulate the formation of crystallites during the secretory stage of tooth enamel development and are thought to play a major role in the structural organization and mineralization of the developing enamel [15].

In young or immature enamel, amelogenin antibodies immunostain 90–95% of the enamel protein. This protein is biosynthesized in young ameloblasts, is secreted into the extracellular enamel matrix [16], and is eventually almost completely removed by extracellular enzymatic degradation during enamel maturation [17].

**Figure 4:**
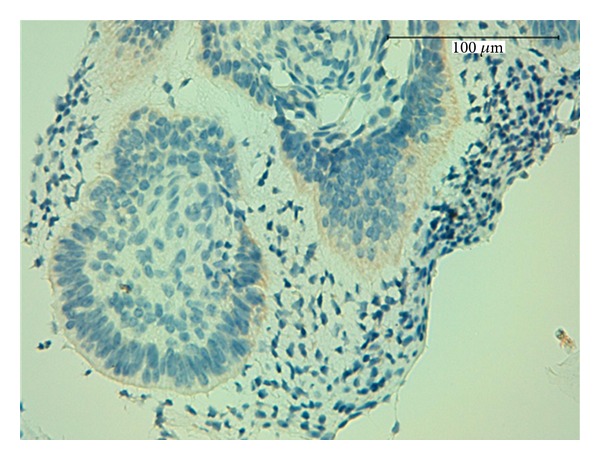
Weak immunopositivity for amelogenin within the peripheral layer of columnar epithelial odontogenic cells, 400x.

Mori et al. [16] reported that epithelial cells stained positively for AMEL, and recently, Crivelini et al. found a strong positive immunoreactivity for AMEL within the distal ends of the epithelial cells of the ameloblastic fibromas and a weaker immunoreactivity in the stellate reticulum-like cells. Consistently, we found immunopositivity for AMEL within the peripheral layer of columnar epithelial odontogenic cells of AFDs, confirming the ameloblastic component of these tumors (Figure 4).

 Calretinin is a 29 kDa, calcium-binding protein that belongs to the family of E-F hand proteins, which includes S-100. The E-F hand proteins are characterized by a helix-loop-helix structure, which functions as the calcium-binding site [18].

**Figure 5:**
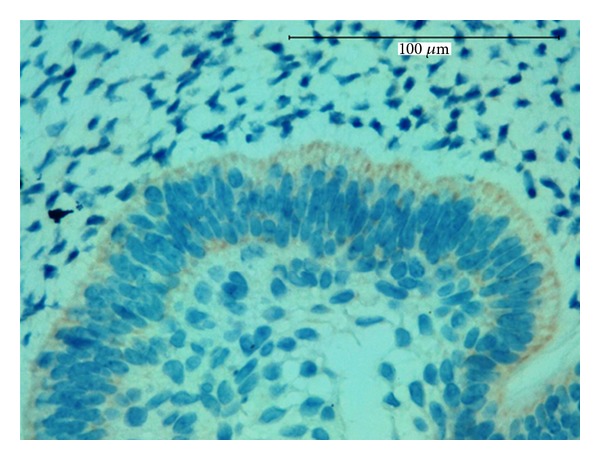
Weak immunopositivity for calretinin antibody in the columnar epithelial odontogenic cells, 600x.

A study by Alaeddini et al. [19] evaluated the expression of calretinin in different odontogenic tumors and did not detect any calretinin expression in ameloblastic fibromas. In contrast, we observed weak immunopositivity for calretinin in the columnar epithelial odontogenic cells of the AFD samples (Figure 5). Mistry et al. [20] demonstrated that calretinin is weakly expressed within the tooth germs of developing rat molars during the early cap stage. They further showed that as the teeth develop, the intensity of the immunoreactivity increases from weak to intense during the late bell stages, indicating that the expression of calretinin in AFD correlates with progressively advanced stages of maturation. Taken together, these data support the theory that all ameloblastic fibromas, AFDs, and ameloblastic fibroodontomas merely represent various progressive stages of the same lesion, ultimately resulting in the formation of odontomas. However, this concept has not been widely accepted for several reasons. For example, a number of cases of recurrent or residual ameloblastic fibromas have demonstrated no evidence of further maturation into a more differentiated odontogenic lesion, such as an ameloblastic fibroodontoma or an odontoma. Moreover, ameloblastic fibromas are known to occur in patient age groups beyond what has been observed for odontogenesis [4, 19, 21]. However, in some studies, for example, in work conducted by Alaeddini et al. [19], they found that ameloblastic fibroma occurred mostly in the third decade; this data also demonstrated that ameloblastic fibroma occurred at a much older age than did the ameloblastic fibroodontoma.

**Figure 6:**
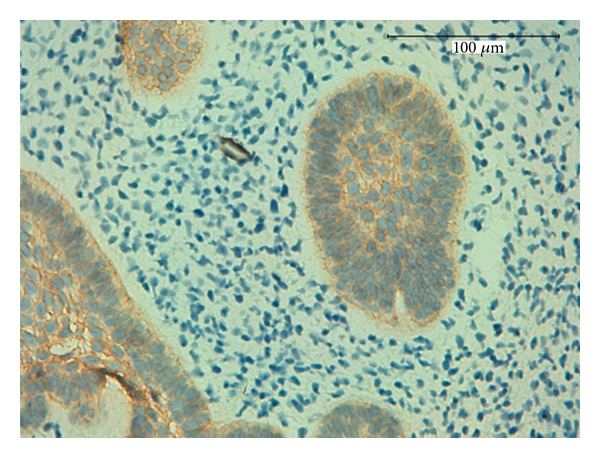
Strong expression for E-cadherin in the islands and the strands of odontogenic epithelium, magnification 400x.

Cadherins (named for “calcium-dependent adhesion”) are a class of type-1 transmembrane proteins. They play important roles in cellular adhesion, ensuring that the cells within tissues are bound together. They have been shown to be involved in many biological processes, morphogenesis, cytoskeletal organization and cell migration, as well as in pathological conditions such as cancer [22]. In cancer, the loss of E-cadherin function through genetic or epigenetic mechanisms has been implicated in the progression and metastasis of numerous malignancies [23]. In our study, we found that the expression of E-cadherin is well conserved among some benign neoplasms and is strong in epithelial odontogenic cells (Figure 6).

Syndecan-1 is a cell surface proteoglycan that facilitates cellular attachment to the extracellular matrix. Its expression is downregulated in many transformed cellular models. The loss of syndecan-1 expression decreases intercellular adherence, as well as attachment to the extracellular matrix. The loss of syndecan-1 expression in ameloblastomas and ameloblastic carcinomas has been demonstrated to correlate with more aggressive biological behaviors (invasion and metastasis) [24–26].

**Figure 7:**
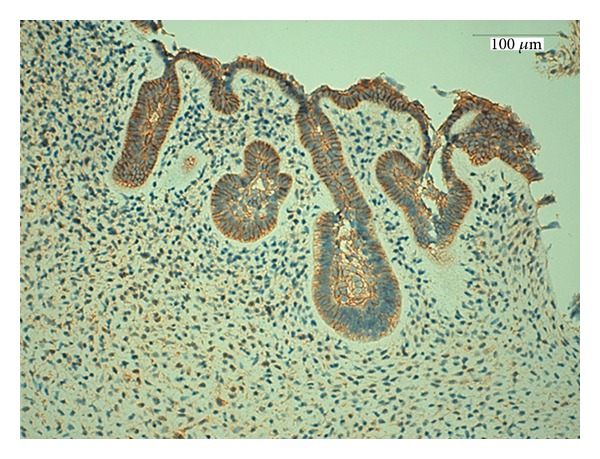
Strong membranous immune positivity for syndecan-1 in the cords and the islands of epithelium and in the central areas resembling the stellate reticulum and moderate positivity in mesenchymal cells, magnification 200x.

In this study, we detected strong membranous immunopositivity for syndecan-1 in the cords and the islands of epithelium and in the central areas resembling the stellate reticulum (Figure 7). The preservation of syndecan-1 expression suggests a cohesion and conservation of the epithelial architecture. Notably, when we evaluated the primitive connective tissue stroma that resembled dental papilla, it exhibited moderate expression levels of syndecan-1 (Figure 7). Beyond its key role as a cell adhesion molecule, syndecan-1 also participates with the extracellular matrix to promote and to regulate cellular proliferation and growth by interacting with families of growth factors that are linked to heparin [27].

**Figure 8:**
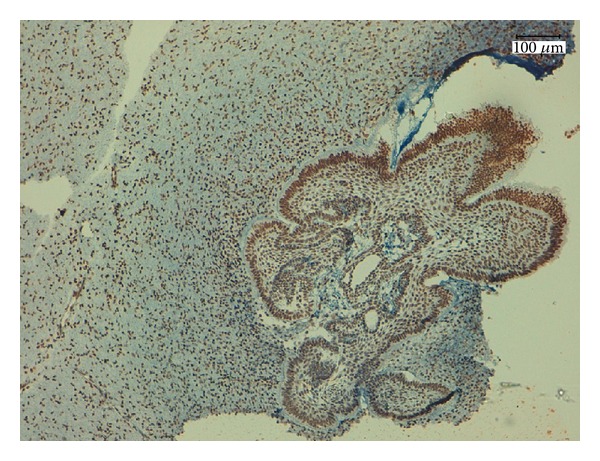
Strong nuclear expression of MSH2 in epithelial and mesenchymal cells, magnification 100x.

The MSH2 gene encodes for an essential DNA repair protein that facilitates the repair of DNA during replication. Immunohistochemical analysis of the expression of MSH2 in tumors indicates that its expression is generally lost in tumors from hereditary nonpolyposis colorectal cancer patients, and the reduced expression of MSH2 has been reported in other types of carcinomas [28]. Leach et al. [29] demonstrated that MSH2 is a ubiquitously expressed protein, exhibiting an exclusively nuclear localization in the normal tissues. We observed strong nuclear expression of MSH2 in ADFs (Figure 8), consistent with a previous study by Castrilli et al. [30], who detected MSH2 expression in all 25 ameloblastomas that they evaluated. These data suggest that the development and progression of these tumors do not depend on a defect in the human DNA mismatch repair system.

**Figure 9:**
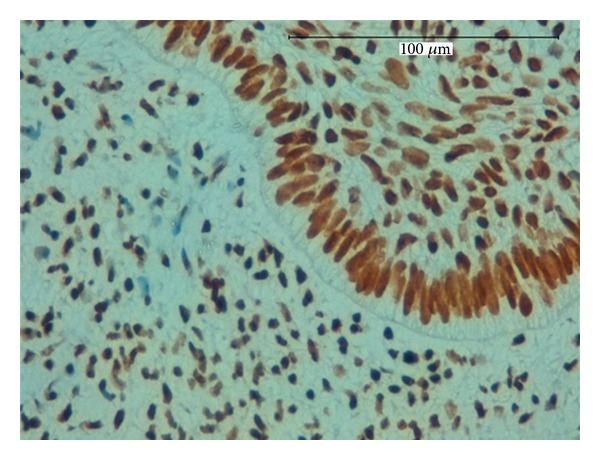
Strong nuclear positivity for the antibody histone H3 in both epithelial and mesenchymal cells, magnification 200x.

**Figure 10:**
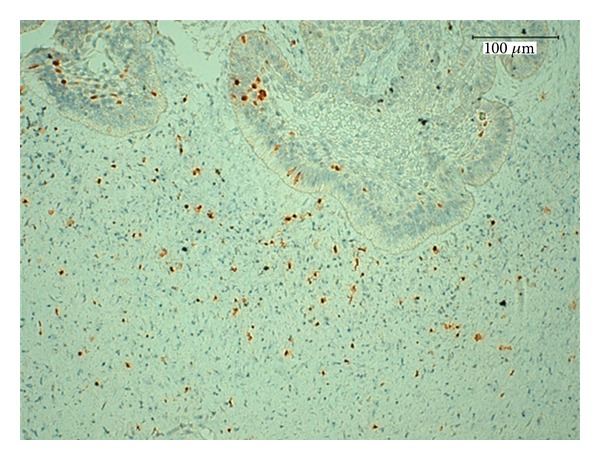
Weak expression of Ki-67 protein in the cords and the islands of epithelium and the mesenchymal cells, magnification 600x.

Analysis of cell proliferation indices in situ provides important insight into the rate of cellular turnover in various tissues or tumors. We evaluated two such cell cycle-related factors, histone H3 and Ki-67, and found that the positively staining cells were distributed in both the epithelial and the mesenchymal compartments. When we compared the immunopositivities of histone H3 and Ki-67, we found that histone H3 exhibited significantly more immunoreactivity in more than half of all epithelial and mesenchymal cells (Figures 9 and 10). These findings suggest that Ki-67 is a more specific proliferation marker for AFD. Moreover, the weak expression of Ki-67 illustrates the low proliferative rate of this tumor, further substantiating the benign nature of this neoplasm. This is consistent with a study by Sano et al., which suggests that the evaluation of growth potential in ameloblastic fibromas and related lesions might enhance our understanding of tumor aggressiveness [31].

## 6. Conclusion

In summary, we have described here a histopathological and immunohistochemical characterization of AFD. In this study, we demonstrated for the first time the presence of these proteins (calretinin, syndecan-1, MSH2, and histone H3) in AFD, some of which can serve as useful markers for understanding the histogenesis and biological behavior of this rare odontogenic tumor.

It is important to keep in mind that due to the rarity of this neoplasm, we have only included one case report. Therefore the interpretation of our results might be limited to a trend or a singular description; future studies should involve a more extensive series of tumor cases to confirm our observations.

## Figures and Tables

**Table 1:** The antibodies used to detect the expression of the different proteins in the epithelial and mesenchymal cells of the AFD samples.

Antibody	Source/clone	Dilution	Antigen retrieval	Epithelial cells	Mesenchymal cells
Amelogenin	Santa Cruz/SC-33109	1 : 100	Mw	+	Neg
CK19	Genetex/polyclonal	1 : 100	Mw	+++	Neg
Calretinin	DAKO/DAK-CALERET 1	1 : 100	Mw	+	Neg
E-cadherin	DAKO/NCH-38	1 : 100	Mw	++	Neg
Syndecan-1	DAKO/MI15	1 : 100	Mw	+++	++
Histone H3	GeneTex/E107	1 : 100	Mw	+++	+++
Ki-67	DAKO/MIB1	1 : 100	Mw	+	+
MSH-2	Genetex/EPR3943	1 : 100	Mw	+++	+++

Neg: negative, +: weak, ++: moderate, +++: strong immunoexpression, and Mw: microwave.
